# Twenty Years of Kaniadakis Entropy: Current Trends and Future Perspectives

**DOI:** 10.3390/e27030247

**Published:** 2025-02-27

**Authors:** Dionissios T. Hristopulos, Sérgio Luiz E. F. da Silva, Antonio M. Scarfone

**Affiliations:** 1 School of Electrical and Computer Engineering, Technical University of Crete, 73100 Chania, Greece; dchristopoulos@tuc.gr; 2 Southern Marine Science and Engineering, 2 University Street, Zhuhai 519082, China; 3 Laboratory of Parallel Architectures for Signal Processing, Federal University of Rio Grande do Norte, Natal 59078-970, RN, Brazil; 4 Istituto dei Sistemi Complessi—Consiglio Nazionale delle Ricerche (ISC-CNR), c/o Dipartimento di Scienza Applicata e Tecnologia del Politecnico di Torino, 10129 Torino, Italy; antoniomaria.scarfone@cnr.it

Napier’s number e=2.7182818284… can be introduced as the limit of a numerical sequence $e=\lim_{n\to \infty }e_{n}$. There are infinite sequences that lead to *e*, the simplest of which was proposed by Euler of the form $e_{n}^{E}={\left(1+1/n\right)}^{n}$. Euler’s sequence converges very slowly and only reaches Napier’s number to two decimal places for n=500. Among the infinite sequences that converge to *e*, there is only one that has the property $e_{-n}=e_{n}$. This new numerical sequence converging toward Napier’s number *e* emerges naturally within Kaniadakis formalism, proposed in a seminal paper from 2001 [1], and has the form

(1) $$e_{n}^{K}={\left(\sqrt{1+\frac{1}{n^{2}}}+\frac{1}{n}\right)}^{n}\;\;.$$

The Kaniadakis sequence converges very quickly to the Napier number, which is already calculated correctly for n=10 to the first two decimal places, while for n=500, the first five decimal places have been obtained correctly.

Starting from the two numerical sequences that lead to the Napier number, it is possible to construct the corresponding sequences of functions that converge to the exponential function. The Euler sequence of functions leading to the exponential function is very simple and is given by $\exp_{n}\left(x\right)={\left(1+x/n\right)}^{n}$. This sequence represents a one-parameter deformation of the ordinary exponential function, with *n* being the deformation parameter. This deformed exponential of Euler’s function $exp_{n}\left(x\right)$ has been used in statistical mathematics to construct the Student distribution, which has been employed to study astrophysical plasmas since the end of the 1960s. This distribution is also known as the κ distribution, and the plasmas described by this distribution are called κ plasmas. The name is derived from the fact that instead of the deformation parameter *n*, parameter κ is historically used so that in the κ→∞ limit, the κ distribution reduces to the corresponding ordinary exponential distribution. Since the late 1980s, Euler deformation with the new parameter q=(1+n)/n has been used instead of *n* in the development of non-extensive statistical mechanics.

Given the impact of the Euler deformation in plasma physics, in statistical physics, and more generally in statistical sciences, in 2001, Kaniadakis introduced a deformation of the exponential function, which now bears his name. The new deformed function captures properties of the ordinary exponential function. The starting point is the property of the self-duality of the ordinary exponential function, that is, exp(x)exp(−x)=1. The deformation parameter is denoted by κ, and the deformed exponential is defined by

(2) $$\exp_{\kappa }\left(x\right)={\left(\sqrt{1+\kappa^{2}x^{2}}+\kappa x\right)}^{1/\kappa }\;\;.$$

In contrast with the homonymous parameter of κ plasmas, for the new deformed exponential $\exp_{\kappa }\left(x\right)$, the range of κ is 0<κ<1. The ordinary exponential is obtained in the limit $\lim_{\kappa \to 0}\exp_{\kappa }\left(x\right)=\exp\left(x\right)$. Like the ordinary exponential function, the Kaniadakis exponential is self-dual, that is, $\exp_{\kappa }\left(x\right)\exp_{\kappa }\left(-x\right)=1$.

All properties of the ordinary exponential function exp(x) are transferred in an identical form to the function $\exp_{\kappa }\left(x\right)$, accordingly generalized by the parameter κ. This makes the function $\exp_{\kappa }\left(x\right)$ particularly interesting for the construction of a statistical theory in analogy to the standard statistical theory of Boltzmann–Gibbs–Maxwell, which is based on the function exp(x). Kaniadakis showed in 2001 [1] that κ-statistical mechanics preserve important features of classical statistical mechanics. The most interesting difference between the two theories is the behavior of the tails of the associated statistical distributions: while the ordinary theory leads to exponential tails, in κ-statistical mechanics, such tails are described by Pareto power laws.

It is noteworthy that power-law tails are also present in Euler’s deformed exponential function and in the related statistical theory. The main difference between the two statistical theories is that Kaniadakis theory arises in the framework of special relativity, as shown in papers from 2002 and 2005 [2,3]. The Kaniadakis deformation is therefore a relativistic effect, which thus provides a robust physical basis for κ-statistical theory.

In the three seminal papers mentioned above, Kaniadakis introduced influential concepts that have transformed our understanding of the physics of complex systems. His pioneering work laid the foundation for what is now known as Kaniadakis entropy or κ-entropy. This concept emerged as a relativistic generalization of the classical Boltzmann–Shannon entropy, marking a significant advance in the framework of special relativity and statistical mechanics. Kaniadakis entropy is defined according to the following:

(3) $$S_{\kappa }=-\sum_{i}f_{i}\ln_{\kappa }\left(f_{i}\right)=\sum_{i}f_{i}\ln_{\kappa }\left(1/f_{i}\right)\;\;,$$

where $f_{i}$ represents the probability distribution function (probability mass function), while $\ln_{\kappa }\left(f\right)$ is the Kaniadakis logarithm, defined as the inverse of the $\exp_{\kappa }\left(x\right)$ function. The summation is over all the states of the system. In the continuum case, the summation is replaced by integration, and $f_{i}$ is replaced by the probability density function f(x).

The κ-deformed logarithm satisfies the *scaling property* $\frac{d}{df}\left[f\ln_{\kappa }\left(f\right)\right]=\frac{1}{\gamma_{\kappa }}\ln_{\kappa }\left(e_{\kappa }f\right)$, where $\gamma_{\kappa }$ and $e_{\kappa }$ are two scaling constants. The maximization of the entropy $S_{\kappa }$, subject to available moment constraints, leads to a maximum entropy probability distribution expressed univocally in terms of the $\exp_{\kappa }\left(x\right)$ function. This extends the exponential maximum entropy distributions obtained by maximizing Shannon’s entropy.

Interestingly, the above scaling property is common to the following three functions: the ordinary logarithm, the Euler-deformed logarithm, and the κ-deformed logarithm. Of course, different scaling constants apply to the three logarithms. However, the self-duality property is only present for the ordinary and κ-deformed functions. The property of self-duality for exp(x) and $\exp_{\kappa }\left(x\right)$, i.e., $\exp_{\kappa }\left(x\right)=1/\exp_{\kappa }\left(-x\right)$, can also be expressed for the inverse functions. If we define $f=\exp_{\kappa }\left(x\right)$ and operate with $\ln_{\kappa }$ on both sides of the self-duality equation, we obtain $\ln_{\kappa }\left(1/f\right)=-\ln_{\kappa }\left(f\right)$ and, in the κ→0 limit, ln(1/f)=−lnf. The above *self-duality* property allows us to interpret the entropy $S_{\kappa }$ as the mean value of the function $-\ln_{\kappa }\left(f_{i}\right)$ or equivalently that of $\ln_{\kappa }\left(1/f_{i}\right)$; thus, κ-entropy extends the definition of Boltzmann entropy while maintaining the self-duality property.

Ordinary statistical mechanics are based on the Boltzmann–Gibbs–Shannon (BGS) entropy or logarithmic entropy. The uniqueness of BGS entropy is derived from the four Khinchin–Shannon axioms, namely continuity (the entropy changes continuously with changes in the probability distribution), maximality (it is maximized by the uniform distribution), expansibility (adding a zero-probability event does not change the entropy), and strong additivity (the entropy of the sum of two independent variables is the sum of the individual entropies). In this Special Issue, Kaniadakis proposes that the Boltzmann–Gibbs–Shannon entropy be introduced by retaining the first three Khinchin–Shannon axioms while replacing the fourth axiom with two new axioms: the so-called *scaling* and *self-duality* axioms that arise naturally in special relativity. The new set of axioms allows for the introduction of ordinary entropy without using the concepts of additivity or statistical independence. Kaniadakis also shows that the new set of axioms is uniquely satisfied by κ-entropy in addition to ordinary entropy; the latter is obtained from κ-entropy in the classical limit κ→0. The emergence of Kaniadakis entropy in special relativity allows us to revisit relativistic thermodynamics problems, providing evidence for the Einstein–Planck proposal of relativistic temperature reduction in a moving frame (see Kaniadakis’ contribution in this Special Issue).

The scientific community has embraced and expanded on the innovative framework of Kaniadakis entropy since its introduction. Hence, the mathematical and practical implications of κ-statistics have been the subject of over 260 papers published by more than 200 scientists. For a comprehensive list of these works, please refer to the References section. The applications of Kaniadakis entropy extend over a wide range of disciplines [1,2,3,4,5,6,7,8,9,10,11,12,13,14,15,16,17,18,19,20,21,22,23,24,25,26,27,28,29,30,31,32,33,34,35,36,37,38,39,40,41,42,43,44,45,46,47,48], including kinetic theory [49,50,51,52,53,54,55,56,57,58,59,60,61,62,63,64], thermodynamics [65,66,67,68,69,70,71], astrophysics [72,73,74,75,76,77,78], cosmology [79,80,81,82,83,84,85,86,87,88,89,90,91,92,93,94,95,96,97,98,99,100,101,102,103,104], dark energy models [105,106,107,108,109,110,111,112,113,114,115,116,117,118,119,120,121,122,123,124,125,126,127], quantum gravity [128,129,130,131,132,133,134,135,136,137,138,139], information geometry [140,141,142,143,144,145,146,147], information theory and optimization [148,149,150], classical statistics [151,152,153,154,155,156,157,158,159,160,161,162,163,164,165,166,167,168,169], quantum statistics [170,171,172,173,174,175], artificial intelligence [176,177,178], dynamical systems [179,180,181,182,183,184,185,186,187,188,189], econophysics, sociophysics and network theory [190,191,192,193,194,195,196,197,198,199,200,201,202,203,204,205,206,207,208,209,210,211,212], geophysics and geomechanics [213,214,215,216,217,218,219,220,221,222,223], genomic analysis [224,225,226,227], nuclear physics [228,229,230,231,232,233,234,235,236], and plasma physics [237,238,239,240,241,242,243,244,245,246,247,248,249,250,251,252,253,254,255,256,257,258,259,260,261,262,263], among other topics. The versatility and applicability of κ-entropy highlight its profound impact on theoretical foundations and practical applications across scientific fields.

This Special Issue aims to capture the current trends and future perspectives of Kaniadakis entropy and its applications:

- Tan et al. (2022) [253] investigate the dispersion and Landau damping of Langmuir and ion acoustic waves in a κ-deformed Kaniadakis distributed plasma system. Their analysis shows that dispersion increased with increasing κ, while Landau damping was suppressed. These results could provide insights into plasma particle trapping and energy transport.
- Biró (2022) [42] explores the use of generalized exponentials in particle–hole symmetry, demonstrating that the Kaniadakis κ-approach is compatible with the Kubo–Martin–Schwinger (KMS) relation, and discusses potential further generalizations.
- De Lima et al. (2022) [226] study the distribution of plant DNA lengths in three Cucurbitaceae species (gourd family). Using Bayesian analysis, they find that the sum of two κ-exponential distributions provides the best fit (among other models) to the empirical distribution curves.
- Hristopulos and Baxevani (2022) [220] introduce a nonlinear normalizing transformation and its inverse based on deformed logarithmic and exponential functions; the transformed pair is useful in the analysis of skewed data, and its inverse is stable, unlike the commonly used Box-Cox transform pair. They also discuss the connection between the heavy-tailed κ-Weibull distribution and the weakest-link scaling theory, showing that the former is suitable for modeling mechanical strength distributions of materials. The paper also introduces the κ-lognormal probability distribution, which has a flexible right tail and can be used to model fluid permeability in random porous media.
- De Abreu et al. (2022) [234] review the application of Kaniadakis entropy to nuclear reactor physics. In particular, they focus on the Doppler broadening effect on neutron cross-sections if the Maxwell–Boltzmann distribution is replaced with the Kaniadakis distribution (which uses the deformed exponential). The authors claim more accurate radiative capture cross-section calculations using the Kaniadakis distribution.
- Guha (2022) [61] uses the Calogero–Leyvraz Lagrangian framework to construct 2D Lotka–Volterra replicator equations and N=2 relativistic Toda lattice systems. The kinetic energy term is deformed using the κ-deformed logarithm, resulting in new formulations of the above systems.
- Luciano (2022) [89] explores recent advances and future challenges of Kaniadakis statistics in gravity and cosmology. He focuses on implications of κ-entropy on cosmological theories and Big Bang nucleosynthesis and uses observational evidence to constrain the κ-parameter.
- Wada and Scarfone (2023) [44] present applications of Kaniadakis distributions to various topics in statistical physics and natural science such as Gompertz functions, the Bloch equation for thermal states, contact density dynamics, and the law of large numbers under κ-addition.
- Martinez and de Abreu (2023) [236] present a review on the use of Kaniadakis entropy in nuclear reactor physics. They generate simulated nuclear data under non-thermal equilibrium conditions using the Kaniadakis distribution to address the limitations of the traditional Maxwell–Boltzmann statistics.
- da Silva et al. (2023) [223] introduce a novel objective function for full-waveform inversion problems, incorporating the Kaniadakis κ-Gaussian distribution and optimal transport theory, to mitigate non-Gaussian noise and phase ambiguity in seismic wave analysis.
- Pistone and Shoaib (2023) [147] propose using a specific case of the Kaniadakis logarithm for the exploratory analysis of compositional data. They show that the affine information geometry derived from Kaniadakis’ algorithm provides a consistent framework for the geometric analysis of compositional data. Moreover, they propose a particular functional form of the κ-divergence.
- Clementi (2023) [211] discusses the application of the κ-generalized distribution in the statistical analysis of income data, highlighting the distribution’s analytical properties, and relationships with other distributions. The paper also comments on the very good agreement of the κ-generalized distribution with empirical data.
- Evangelista and Lenzi (2023) [64] use the H-theorem to examine the dynamics of a system composed of two subsystems that obey nonlinear Fokker–Planck equations. They focus on the behavior of the entropy of the entire system considering subsystems that have (i) identical and (ii) different dynamics.
- Scarfone and Wada (2024) [45] demonstrate that Kaniadakis entropy can become multi-additive under a suitably defined constraint, that is, under the composition of two identically distributed probability distributions.
- Kaniadakis (2024) [46] highlights the importance of κ-entropy in statistical theory, identifying five axioms in κ-statistical theory that provide a solid foundation for understanding entropy in complex systems. This study sheds light on the physical origins of κ-entropy, emphasizing the self-duality and scaling axioms as fundamental elements. Kaniadakis also emphasizes the emergence of κ-entropy in Einstein’s special theory of relativity and introduces relativistic statistical mechanics based on the new entropy. Finally, Kaniadakis shows that the new formalism allows us to re-discuss in a modern way some remaining open problems of relativistic thermodynamics introduced by Planck concerning the transformation law of temperature and entropy.

The interdisciplinary collaboration of experts in different fields has substantiated the theoretical foundations of Kaniadakis entropy and established practical applications of the κ-entropy as well as other κ-deformed functions in various scientific fields. This Special Issue celebrates the insightful contributions of Giorgio Kaniadakis and the work of scholars who have significantly advanced the field of κ-statistics.

In addition to presenting a tribute to past achievements and accomplishments, this Special Issue aims to provide motivation and directions for future research. In this spirit, this introduction includes an extensive list of published research on κ-statistics and their applications. The list of references is organized in sections according to the scientific field of application. We believe that this information will be useful for researchers entering the field of κ-statistics.

**Papers published in this Special Issue**
  - Biró, T.S. *Kaniadakis Entropy Leads to Particle–Hole Symmetric Distribution*, Entropy **2022**, 24 (9), 1217. (Ref. [42])
  - Clementi, F. *The Kaniadakis Distribution for the Analysis of Income and Wealth Data*, Entropy **2023**, 25(8), 1141. (Ref. [211])
  - da Silva, S.L.E.F.; de Araújo, J.M.; de la Barra, E.; Corso, G. *A Graph-Space Optimal Transport Approach Based on Kaniadakis κ-Gaussian Distribution for Inverse Problems Related to Wave Propagation*, Entropy **2023**, 25, 990. (Ref. [223])
  - de Abreu, W.V.; Maciel, J.M.; Martinez, A.S.; Gonçalves, A.D.C.; Schmidt L. *Doppler Broadening of Neutron Cross-Sections Using Kaniadakis Entropy*, Entropy **2022**, 24(10), 1437. (Ref. [234])
  - de Lima, M.M.F.; Anselmo, D.H.A.L.; Silva, R.; Nunes, G.H.S.; Fulco, U.L.; Vasconcelos, M.S.; Mello, V.D *A Bayesian Analysis of Plant DNA Length Distribution via κ-Statistics*, Entropy **2022**, 24, 1225. (Ref. [226])
  - Evangelista, L.R.; Lenzi, E.K. *Nonlinear Fokker–Planck Equations, H-Theorem and Generalized Entropy of a Composed System*, Entropy **2023**, 25, 1357. (Ref. [64])
  - Guha, P. *The κ-Deformed Calogero-Leyvraz Lagrangians and Applications to Integrable Dynamical Systems*, Entropy 2023, 24, 1673. (Ref. [61])
  - Hristopulos, D.T.; Baxevani A. *Kaniadakis Functions beyond Statistical Mechanics: Weakest-Link Scaling, Power-Law Tails, and Modified Lognormal Distribution*, Entropy **2022**, 24(10), 1362. (Ref. [220])
  - Kaniadakis, G. Relativistic Roots of κ-Entropy. *Entropy*. **26**, 406 (2024). (Ref. [46])
  - Luciano G.G. *Gravity and Cosmology in Kaniadakis Statistics: Current Status and Future Challenges*, Entropy **2022**, 24(12), 1712. (Ref. [89])
  - Martinez A.S.; de Abreu W.V. *The Scientific Contribution of the Kaniadakis Entropy to Nuclear Reactor Physics: A Brief Review*, Entropy **2023**, 25(3), 478. (Ref. [236])
  - Pistone G.; Shoaib M. *Kaniadakis’s Information Geometry of Compositional Data, Entropy* **2023**, 25(7), 1107. (Ref. [147])
  - Scarfone A.M., Wada T. *Multi-Additivity in Kaniadakis Entropy*, Entropy **2024**, 26(1), 77. (Ref. [45])
  - Tan, L.; Yang, Q.; Chen, H.; Liu S. *The Longitudinal Plasma Modes of κ-Deformed Kaniadakis Distributed Plasmas Carrying Orbital Angular Momentum*, Entropy **2022**, 24 (9), 1211. (Ref. [253])
  - Wada T., Scarfone A.M. On the Kaniadakis Distributions Applied in Statistical Physics and Natural Sciences, Entropy **2023**, 25(2), 292. (Ref. [44])
